# Essential Oil Composition of *Artemisia herba-alba* from Southern Tunisia

**DOI:** 10.3390/molecules14041585

**Published:** 2009-04-20

**Authors:** Haouari Mohsen, Ferchichi Ali

**Affiliations:** Laboratoire d'Aridoculture et Culture Oasienne, Institut des Régions Arides 4119 Medenine, Tunisia; E-mail: Ferchichi.ali@ira.rnrt.tn (F.A.)

**Keywords:** Herba-alba, Asteraceae, Essential oil composition, Chemotypes

## Abstract

The composition of the essential oil hydrodistilled from the aerial parts of 18 individual *Artemisia herba-alba* Asso. plants collected in southern Tunisia was determined by GC and GCMS analysis. The oil yield varied between 0.68% v/w and 1.93% v/w. One hundred components were identified, 21 of of which are reported for the first time in *Artemisia herba-alba* oil. The oil contained 10 components with percentages higher than 10%. The main components were cineole, thujones, chrysanthenone, camphor, borneol, chrysanthenyl acetate, sabinyl acetate, davana ethers and davanone. Twelve samples had monoterpenes as major components, three had sesquiterpenes as major components and the last three samples had approximately the same percentage of monoterpenes and sesquiterpenes. The chemical compositions revealed that ten samples had compositions similar to those of other *Artemisia herba-alba* essential oils analyzed in other countries. The remaining eight samples had an original chemical composition.

## 1. Introduction

*Artemisia herba-alba* Asso. known also as "desert wormwood" is a prominent plant of the Irano-Turanien steppes of Spain, North Africa and the Middle East [1,2,3,4]. It is one of five spontaneous *Artemisia* species recorded in Tunisia [5]. This plant is used as aromatisant for tea and in folk medicine for treatment of colds, coughing, intestinal disturbances and as antidiabetic agent [6,7]. A survey carried out among the South Tunisian population showed that *Artemisia herba-alba* is the most recognized aromatic and medicinal plant [8]. In Tunisia, this greenish-silver perennial shrub is present in areas with between 400 mm and 90 mm of annual precipitations. It is characterized by its drought tolerance, essentially due to its leaves’ polymorphism and root architecture [9,10,11].

Investigations on the medicinal properties of *A. herba-alba* extracts reported anti-diabetic, leishmanicidal, antibacterial, and antifungal properties [12,13,14,15]. Over last decades, studies on *A. herba-alba* were focused on its essential oils. Their composition in different parts of the world revealed a high level of polymorphism and led to the definition of several chemotypes.

Studies from Spain [16,17] showed that monoterpene hydrocarbons and oxygenated monoterpenes are the most abundant skeletons in *A. herba-alba* oil, but large amounts of sesquiterpenes were found for some populations. Camphor, 1,8-cineole, *p*-cymene and davanone were the major compounds found. Two oil types were found for plants grown in Israel and Sinai [18] those of cineol-thujane-bornane type and the pinane type with monoterpene skeletons. Chemotaxonomic affinity between Spanish and Israeli populations of the same plant was not reflected in their oil composition.

In Jordan regular monoterpenes were predominant and the principal components were α- and β-thujones, classifyng the plant as being a thujone chemotype [19]. In Morocco, the market leader in *A. herba-alba* essential oil exports, 16 chemotypes were found [20], with 12 having monoterpenes as major components and for four, sesquiterpene skeletons represent the major fraction of the oil. Investigations reported no correlation between chemotypes and geographic distribution. [21]

For Algerian oil monoterpenes were the major components, essentially camphor, α- and β-thujones, 1,8-cineole and chrysanthenyl derivatives [22,23]. In Tunisian oil oxygenated monoterpenes were found to be the major components of *A. herba-alba* oil extracted from aerial parts of plants originated from arid regions [24,25].

Studies on *A. herba-alba* didn’t cover the entire domain where this plant is found. The aim of this study was to elucidate the chemical polymorphism of *A. herba-alba* from the sub-arid to Saharan domains in Tunisia.

## 2. Results and Discussion

In order to study the chemical composition and variability of the *A. herba-alba* essential oils, 18 individual plants were collected from different populations located in Southern Tunisia. These plants were selected from environmentally diverse areas and subcultured under the same ecological conditions.

Hydrodistillation of the aerial parts of these *A. herba-alba* samples yielded yellowish liquid oils. The oil yield varied between 0.68% v/w and 1.93% v/w, based on dried weight of samples. Same variation in the essential oil yield was also found in Spain [17].

The chemical composition of the oil was investigated using both GC and GC-MS techniques. Table 1 lists the components identified in the essential oil of *A. herba-alba*, their RI, and their concentration in the different samples. One hundred components, accounting for 93.5% to 100% of the oil, were identified, 75 of them being reported for the first time in Tunisian oil, to our knowledge, and 21 of which have not been previously reported in *A. herba-alba* oils (see footnotes in Table 1). Among the 18 essential oil samples, monoterpenes constituted the major fraction of the oil in 12 samples, amounting to more than 57% of the total oil (Table 1). In three samples (E2, E10, and E14), sesquiterpenes were more abundant than monoterpenes, with concentrations as high as 54% of the total oil. For the last three samples (E3, E6, and E13), monoterpenes and sesquiterpenes have sensibly the same contribution to the total oil composition (Table 1). For monoterpene-rich samples, oxygenated monoterpenes are the most abundant and for sesquiterpene-rich samples, oxygenated sesquiterpenes represent the major fraction of the oil.

Among the hundred identified components, ten of them (cineole, thujones, chrysanthenone, camphor, borneol, chrysanthenyl acetate, sabinyl acetate, davana ethers and davanone) could be considered as major components, with concentrations superior to 10% of the total oil.

Cineole was reported as the major component in the essential oil from Spain [16,17], Israel [18], Egypt [26], and Morocco [20]. Its concentration reaches high values (superior to 40%) in Spain and Israel [17,18]. In Tunisian *A. herba-alba* oil, previous studies reported none or very low values for 1,8-cineole concentration [24,25], like essential oils from Algeria and Jordan [19,23]. In this study 1,8-cineole concentration varied between 0.6% in sample E2 and 26% in sample E18. This kind of variation was observed in Moroccan oil where a cineole-camphor chemotype was defined [20].

Thujones were absent in two samples (E2 and E3) but represented 64% of the E15 sample. These components give the specific odor and taste to *A. herba-alba* plants. The absence of thujones was reported for all the oils from Spain [16,17]. Reported concentration in the other oils varies, but didn’t reach the concentration observed for the E15 sample in this study.

Chrysanthenone reached the highest percentage (17%) in sample E2. This component, with a pinane skeleton, wasn’t found in Tunisian *A. herba-alba* essential oil at such high concentrations [24,25]. In Spain and Algeria, chrysanthenone was found at amounts comparable to those found in this study [17,23]. In Morocco, a chrysanthenyl chemotype was defined, with chrysanthenone concentrations as high as 77% of the total oil [20].

Camphor was found at high concentrations in five samples (E1, E11, E12, E17, and E18) and constituted the major component in two of them (E1 and E12). This chemical is one of the most encountered components in *A. herba-alba* oil. In Morocco, five chemotypes were defined as camphor type oils [20].

Borneol was found as a major component in sample E18. High levels of borneol were found in oils from the Sinai [18], but the chemotype defined there (cineole-thujone-borneol) is characterized by its high level of thujone, whereas sample E18 has a low level of thujone but a high camphor concentration.

Chrysanthenyl acetate was found at its highest concentrations in sample E9 with sabinyl acetate and thujones as main components. Such association was observed in *A. herba-alba* from Tunisia [24].

Sabinyl acetate was present as a major component in five samples, with a concentration range from 10% to 22% of the total oil. High levels of sabinyl acetate were only found in oils originated from the south of Tunisia [8,24,27].

The main sesquiterpenes found in the studied oils are davana ethers and davanone. Several davana ether isomers were identified. In Tunisia (this study) three isomers were found, while in Spain two isomers were identified [17], and in Morocco four isomers were described in *A. herba-alba* oil [20].

Davanone, and its derivatives, were also identified in Israel [18], but the highest levels were observed in Morocco, leading to the definition of four davanone chemotypes [20].

Of the 18 samples studied ten have compositions similar to published essential oils of *A. herba-alba*. Sample E4 has thujones and sabinyl acetate as main components as the oil extracted from other locations in Tunisia [24]. Samples E6, E7, E8, E14, E15, and E16, with thujones as major components, have their equivalent in Moroccan and Jordanian oils [19,20]. A thujones-camphor oil (sample E12) was also observed in Morocco [20] and cineole-camphor-borneol oil (sample E18) was observed in Israel [18]. The most frequent chemotype is composed of cineol and camphor and was observed in sample E17 and in Morocco, Spain and Israel [16,18,20].

The eight remaining samples (E1, E2, E3, E5, E9, E10, E11, and E13) have an original composition. The main components association observed in these oils was not observed in *A. herba-alba* essential oil composition published elsewhere.

**Table 1:** Chemical composition of essential oils of *Artemisia herba-alba*.

Name	RI	E1	E2	E3	E4	E5	E6	E7	E8	E9	E10	E11	E12	E13	E14	E15	E16	E17	E18
2-Hexanal^a^	841	0.22	-	0.28	0.35	0.27	-	0.2	0.23	-	0.28	0.53	0.22	-	-	0.74	-	0.36	-
*n*-Hexanol^a^	860	-	-	0.26	-	-	-	-	-	-	0.23	-	-	-	-	-	-	-	-
Geraniolene^a^	880	-	-	-	-	-	-	0.13	-	-	0.13	-	-	-	-	-	-	-	-
Santolina triene	909	-	-	0.23	-	0.21	-	-	-	-	0.14	-	-	0.14	-	-	-	0.52	-
2.5-Diethenyl-2-methyltetrahydrofuran	916	-	0.39	0.47	0.28	0.53	0.32	-	0.33	0.21	0.35	-	-	0.36	-	-	0.15	-	-
Tricyclene	924	-	-	-	-	-	-	-	-	-	-	0.28	0.29	-	-	-	-	0.31	0.34
α-Pinene	933	0.2	-	0.27	-	0.16	0.17	0.35	0.61	-	0.22	-	0.62	-	0.13	-	0.34	-	-
Unknown 1	940	-	0.43	0.54	0.37	0.65	0.42	-	0.33	-	0.38	-	-	0.46	-	-	0.22	-	-
Camphene	949	1.64	0.28	0.31	-	0.25	0.95	1.44	0.51	0.26	0.35	2.42	2.45	-	-	-	0.34	2.04	2.08
5.5-Dimethyl-2(5H)-furanone	952	-	0.37	0.59	-	-	-	-	-	-	-	-	-	-	0.44	-	-	-	-
Sabinene	971	-	0.17	0.12	0.67	-	0.2	-	0.4	-	0.17	-	1.5	0.48	-	1.14	0.51	-	-
β-Pinene	978	1.15	-	-	-	0.91	-	-	-	-	-	-	0.42	-	-	-	-	-	-
1-Octen-3-ol	983	-	-	-	-	-	-	-	-	-	-	-	-	-	-	-	-	-	1.03
β-Myrcene	994	-	-	0.42	-	0.4	-	0.56	-	-	0.46	-	0.4	-	-	-	0.5	-	-
α-Phellandrene	998	-	-	-	-	-	-	-	-	-	-	-	-	-	-	-	0.21	-	-
Yomogi alcohol	999	-	-	-	-	0.18	-	-	-	-	-	-	-	-	-	-	-	3.72	9.46
2-Carene^a^	1001	1.16	-	-	-	0.2	-	-	-	-	-	-	-	-	-	-	-	-	-
α-Terpinene	1012	-	-	-	-	-	-	-	0.23	-	-	-	0.34	-	-	0.53	0.29	-	-
*p*-Mentha-1(7).8-diene	1018	-	0.25	0.31	-	0.36	-	-	-	-	0.25	-	-	0.25	-	-	-	-	-
*p*-Cymene	1025	-	-	0.35	0.79	0.7	0.67	2.3	0.85	0.82	-	2.18	-	0.65	0.3	2.77	1.39	-	-
Eucalyptol	1032	11.18	0.61	2.68	2.58	2.14	4.88	5.22	2.46	4.24	1.52	10.19	6.89	1.57	1.07	7.33	4.13	18.35	26.99
Santolina alcohol	1038	-	-	-	-	-	-	-	-	-	-	-	-	-	-	-	-	3.71	3.37
*cis*-Arbusculone	1052	-	2.98	4.24	-	-	-	1.96	2.81	1.09	4.58	-	-	1.75	0.91	-	0.9	-	-
γ-Terpinene	1062	1.92	-	0.28	0.48	0.45	0.22	-	0.42	-	-	-	0.67	0.31	-	1.2	0.4	0.47	0.53
Artemisia ketone	1064	-	-	-	-	-	-	-	-	-	-	-	-	-	-	-	-	2.65	-
*cis*-Sabinene hydrate	1070	2.08	-	-	-	-	-	-	-	-	-	-	-	-	-	-	-	-	-
*trans*-Arbusculone	1072	-	2.27	3.53	-	-	-	1.48	2.35	0.92	3.6	-	-	1.65	0.72	-	0.75	-	-
Artemisia alcohol	1084	-	-	-	-	-	-	-	-	-	-	-	-	-	-	-	-	2.05	2.97
Terpinolene	1088	0.75	-	-	-	-	-	-	-	-	0.28	-	-	-	-	-	-	-	-
Isochrysanthenone	1098	-	2.95	2.09	-	-	-	-	-	-	3.96	-	-	-	-	-	-	-	-
α-Thujone	1110	11.54	-	-	34.32	8.2	12.85	20.04	5.99	16.38	0.8	23.16	16.51	11.63	13.39	42.23	11.36	4.58	2.11
β-Thujone	1114	6.03	-	-	4.41	5.48	2.18	10.88	6.96	9.57	-	24.05	11.18	5.88	5.06	22.44	3.4	5.74	1.28
*cis-p*-Menth-2-en-1-ol	1118	2.19	-	-	0.77	-	-	-	1.04	-	-	-	-	0.6	-	-	-	2.15	-
Chrysanthenone	1123	-	17.37	7.89	-	10.08	4.83	8.97	1.07	9.02	11.65	1.15	8.64	-	6.19	0.91	8.34	2.69	3.8
*trans*-Pinocarveol	1136	-	0.63	0.9	0.56	0.34	-	-	5.39	2.68	0.62	-	-	0.7	2.76	1.79	2.58	-	-
δ-Verbenol^a^	1141	-	0.68	4.58	-	-	-	-	-	2.12	0.34	-	-	-	-	-	3.2	-	-
*cis*-Sabinol	1143	-	-	-	5.94	1.64	-	-	2.4	-	-	-	-	2.49	-	-	-	-	-
Camphor	1144	13.25	-	-	0.56	-	5.96	7.05	-	-	0.63	16.73	17.81	-	0.61	2.54	-	17.13	12.85
Ssabina ketone	1156	-	-	-	-	-	-	-	-	0.37	-	0.73	-	0.24	-	0.53	-	-	-
Pinocarvone	1160	1.07	-	-	-	-	0.32	0.4	0.68	0.41	-	0.77	1.2	-	0.39	0.83	0.73	0.91	0.95
Borneol	1168	1.91	-	0.72	-	-	2.14	1.61	1.13	0.89	-	4.11	5.24	-	0.55	0.69	0.91	4.94	10.75
α-Phellandren-8-ol^a^	1170	-	-	1.17	-	-	-	-	-	-	-	-	-	-	-	-	-	-	-
Lavandulol	1170	-	-	-	-	-	-	-	-	-	-	-	-	-	-	-	-	1.44	-
4-Terpineol	1177	8.71	0.98	0.89	3.38	1.93	1.7	1.81	2.12	0.57	1	1.5	2.65	1.24	1.75	3.4	1.7	2.5	2.08
α-Thujenal^a^	1182	0.72	-	-	-	-	-	-	0.33	-	-	-	-	0.4	-	-	0.43	-	-
*p*-Cymen-8-ol	1184	-	-	-	-	-	-	-	-	-	-	-	-	-	-	-	-	-	1.14
α-Terpineol	1189	1	-	-	-	-	-	-	-	-	-	-	0.62	-	0.31	-	-	-	1.02
*cis*-Piperitol	1194	-	-	-	-	-	-	-	-	0.98	-	-	-	-	-	-	1.37	-	-
Myrtenal	1197	-	-	-	-	-	-	-	-	-	-	0.73	-	-	-	-	-	1.26	1.21
Myrtenol	1198	1.21	-	-	-	-	0.45	-	1.08	-	-	0.79	1.11	-	0.67	1.27	-	0.92	1.21
Verbenone	1203	-	-	0.37	-	-	-	-	-	-	-	-	-	-	-	-	-	-	-
*trans*-Piperitol	1211	1.15	-	-	-	0.71	0.46	0.69	0.63	1.19	-	-	1.28	0.46	0.58	0.62	1.67	-	-
Octyl acetate^a^	1216	-	-	-	-	-	-	-	-	-	-	-	-	-	-	-	-	-	1.11
Nordavanone	1227	-	0.59	0.35	-	0.54	0.48	-	0.46	-	-	-	-	0.78	-	-	-	-	-
Cuminaldehyde	1235	1.63	-	-	-	-	-	-	0.62	-	-	-	-	0.73	-	-	0.72	-	-
Butanoic acid, 2-methyl- hexyl ester^a^	1237	-	-	-	-	-	-	-	-	-	0.24	-	-	-	-	-	-	-	-
Carvone	1239	-	-	-	-	-	-	-	-	-	-	-	-	-	0.31	-	-	0.65	-
Piperitone	1253	-	-	-	-	-	-	-	0.66	4.56	-	-	-	0.75	-	0.81	3.43	1.83	-
Piperitone oxide^a^	1259	-	-	-	-	-	-	-	0.6	-	-	-	0.65	0.6	0.33	-	1.22	-	-
*cis*-Chrysanthenyl acetate	1262	1.08	0.52	9.47	-	9.11	3.61	2.58	5.54	13	-	1.12	5.82	0.37	1.01	0.62	7.37	0.88	2.28
Isopiperitenone^a^	1271	-	-	0.24	-	-	-	-	-	-	0.39	-	-	-	-	-	-	-	-
Bornyl acetate	1285	0.71	-	-	-	-	-	0.81	0.43	-	-	0.76	1.27	-	0.38	-	-	1.01	2.04
4-Terpineol acetate	1289	-	-	-	-	-	-	0.53	-	-	-	-	-	-	-	-	-	-	-
Sabinyl acetate	1297	12.12	-	-	22.46	10.98	7.36	3.13	6.52	11.09	0.53	1.12	-	11.13	-	-	8.23	4.16	1.02
Myrtenyl acetate	1302	0.73	-	0.27	-	-	-	-	-	-	-	-	-	-	-	-	-	-	-
Thymol	1289	-	-	-	-	-	-	0.52	0.39	-	0.34	-	-	-	-	-	0.37	-	-
Carvacrol	1305	0.59	-	-	-	-	-	-	-	-	-	-	-	-	-	-	0.57	-	-
Unknown 2	1310	-	-	0.34	-	0.4	-	0.83	-	-	0.6	-	-	-	-	-	0.49	-	-
α-Copaene	1376	-	-	-	-	-	0.44	0.68	-	-	-	-	-	-	0.31	-	-	-	-
Methyl cinnamate^a^	1379	0.77	-	-	-	-	-	-	0.54	-	-	-	-	-	-	-	-	-	-
Jasmone	1394	0.79	2.25	0.69	0.83	1.11	0.88	1.75	0.87	-	0.95	-	0.95	0.9	1.64	0.75	-	-	-
*cis*-Davanafuran	1414	-	-	-	-	-	-	-	0.43	-	-	-	-	0.49	-	-	-	-	-
α-Humulene	1454	-	-	-	-	-	-	1.03	-	-	1.14	-	-	-	-	-	-	-	-
Ethyl cinnamate	1464	2.47	-	-	-	-	-	1.82	-	-	-	3	-	-	-	1.03	-	2.12	-
Germacrene D	1474	4.15	2.92	2.37	5.63	2.85	6.67	3.07	4.82	2.11	2.59	2.04	5.29	4.13	5.93	2.32	2.22	3.05	-
β-Selinene	1476	-	0.46	-	-	0.71	-	0.64	0.65	0.71	-	-	-	-	1.15	-	0.48	-	-
Lyratyl isovalerate^a^	1478	-	-	1.16	-	-	-	-	-	0.58	-	-	-	-	-	-	-	-	-
Bicyclogermacrene	1482	2.87	2.21	1.71	4.05	2.2	6.16	1.46	2.69	2.08	3.04	1.18	2.56	2.44	5.63	2.39	1.53	1.95	1.48
Davana ether	1491	-	4.43	4.56	0.65	1.09	1.21	0.82	2.46	0.62	6.23	-	-	3.02	2.55	-	1.59	-	-
Unknown 3	1494	-	-	-	0.8	0.44	-	-	0.38	-	-	-	-	0.54	-	-	-	-	-
Davana ether isomer	1523	-	12.53	11.59	1.34	3.24	3.71	1.97	6.61	1.72	15.94	-	-	8.42	5.83	-	4.69	-	-
Davana ether isomer	1554	-	6.62	6.13	-	1.65	1.44	0.81	3.41	0.85	7.27	-	-	4.5	2.87	-	2.4	-	-
Nerolidol	1570	-	-	0.9	-	-	-	-	-	-	0.91	-	-	-	3.46	-	-	-	-
Spathulenol	1576	1.09	1.12	-	0.96	1.28	3.74	3.74	0.99	1.39	0.55	1.36	1.02	1.42	3.1	0.99	0.85	1.9	2.06
Globulol^a^	1583	-	-	-	-	-	1.41	0.64	0.55	-	-	-	-	0.48	1.77	-	-	-	-
Caryophyllene oxide	1587	-	-	-	-	-	-	-	-	-	1.03	-	-	-	-	-	-	-	-
β-Copaen-4-α-ol^a^	1590	-	-	0.8	-	-	-	-	-	-	0.45	-	-	-	-	-	1.05	-	-
Epiglobulol^a^	1593	0.69	-	-	-	-	2.08	-	-	-	-	-	0.57	-	0.82	-	-	-	-
Davanone	1608	-	20.14	11.03	6.3	16.94	7.95	-	7.07	2.37	9.82	-	-	13.37	4.02	-	6.35	-	-
Rosifoliol^a^	1611	-	-	-	-	-	-	-	-	-	-	-	-	-	1	-	-	-	-
3-Methyl-but-2-enoic acid, 1.7.7-trimethylbicyclo[2.2.1]hept-2-yl ester^a^	1619	-	-	-	-	-	0.94	-	-	-	-	-	-	0.74	-	-	-	-	-
α-Acorenol	1622	-	-	0.57	-	0.69	1.09	0.94	-	-	0.44	-	0.95	-	2.48	-	0.6	-	-
τ-Cadinol	1638	-	-	-	-	0.53	1.42	1.3	-	-	-	-	0.74	-	3.46	-	-	-	-
3-Hydroxyisodavanone	1644	-	4.96	5.23	-	1.06	1.12	2.5	4.8	4.74	5.41	-	-	4.39	2.97	-	4.29	-	-
β-Eudesmol	1650	-	-	-	-	-	0.97	0.68	-	-	-	-	-	-	-	-	-	-	-
Unknown 4	1655	-	-	-	-	-	-	-	1.73	-	-	-	-	-	2.77	-	-	-	-
α-Cadinol^a^	1657	0.57	1.67	1.39	-	1.67	1.68	1.1	1.53	-	1.41	-	-	2.45	1.54	-	1.09	-	-
Selin-11-en-4-α-ol	1659	-	-	-	-	-	-	-	1.09	-	-	-	-	-	1.56	-	-	-	-
Xanthoxylin	1661	-	-	-	-	0.74	-	1.22	-	-	-	-	-	-	-	-	0.58	2.13	-
(E)-Dihydrofarnesol^a^	1672	-	-	-	-	1.12	0.85	-	-	-	-	-	-	0.64	-	-	-	-	-
Unknown 5	1679	-	-	-	-	-	0.58	-	-	-	-	-	-	-	0.59	-	-	-	-
Unknown 6	1681	-	3.37	3.11	-	1.75	1.46	-	1.61	-	4.22	-	-	2.18	0.87	-	1.13	-	-
1.2-Dehydro-3-hydroxy-isodavanone^a^	1692	-	3.56	2.37	1.38	2.89	2.12	-	1.52	1.69	2.11	-	-	3.46	0.79	-	1.88	-	-
β-Savanone-2-ol	1722	-	0.79	-	-	0.87	-	-	-	-	-	-	-	-	0.84	-	-	-	-
Phytol^a^	2112	0.52	-	-	-	-	-	-	-	-	-	-	-	-	0.52	-	-	-	-
Total		99.86	99.36	97.77	99.86	99.65	99.22	99.66	99.75	99.23	98.57	99.9	99.86	99.81	97.55	99.87	98.95	98.12	95.16
Total identified		99.86	95.56	93.78	98.69	96.41	96.76	98.83	95.7	99.23	93.37	99.9	99.86	96.63	93.32	99.87	97.11	98.12	95.16
Monoterpenes hydrocarbons		6.82	0.7	2.29	1.94	3.64	2.21	4.78	3.02	1.08	2	4.88	6.69	1.83	0.43	5.64	3.98	3.34	2.95
Oxygenated monoterpenes		82.93	32.59	41.14	76.09	53.71	48.42	72.47	53.83	79.29	31.5	89.91	81.82	44.72	39.07	87.79	64.11	87.52	88.67
Total monoterpenes		89.75	33.29	43.43	78.03	57.35	50.63	77.25	56.85	80.37	33.5	94.79	88.51	46.55	39.5	93.43	68.09	90.86	91.62
Sesquiterpenes hydrocarbons		7.02	6.45	4.08	9.68	5.76	14.4	6.88	8.59	4.9	7.79	3.22	7.85	7.19	14.24	4.71	4.23	5	1.48
Oxygenated sesquiterpenes		2.35	55.82	45.73	10.63	33.03	31.73	14.5	30.03	13.96	51.57	1.36	3.28	42.89	39.06	0.99	24.79	1.9	2.06
Total sesquiterpenes		9.37	62.27	49.81	20.31	38.79	46.13	21.38	38.62	18.86	59.36	4.58	11.13	50.08	53.3	5.7	29.02	6.9	3.54

^a^components reported for the first time in *A. herba-alba* oils

**Figure 1:**
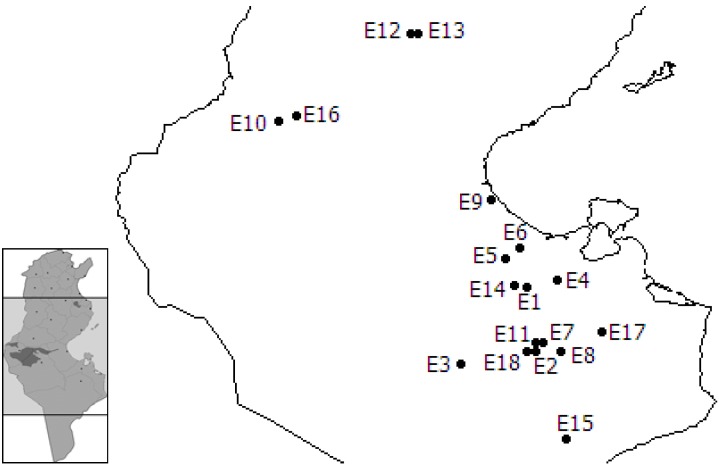
Origins of *Artemisia herba-alba* plants.

## 3. Experimental

### 3.1. Plant material

*A. herba-alba* aerial parts (leaves and stems) were collected from subcultured plants originated from different localities in sub-arid to Saharan domains of Tunisia (Figure 1) at the Institut des Régions Arides Médenine. Plant identification was carried out by A. Ferchichi, botanist at the Institut des Régions Arides Médenine. All samples were shade dried for 15 days at room temperature with ventilation. The material was cut into small pieces and subjected to hydrodistillation using a Clevenger-type apparatus for 4 hours. The oil was collected and stored at -12 °C in amber vials before analysis.

### 3.2. GC analysis

Analytical gas chromatography was carried out using an Agilent-HP6890 gas chromatography system fitted with a DB-5 fused silica capillary column (30 m x 0.32 mm, 0.25 µm film thickness). Carrier gas was helium at a flow rate of 1 mL/min. Column temperature was initially kept at 60°C for 5min, then gradually increased to 280°C at a rate of 2°C/min, and held for 10 min. Samples (1µL, appropriately diluted in hexane) were injected at 280°C with split mode (1:50). A hydrocarbon mixture for retention index (RI) measurement was injected at the same above conditions. Flame ionization detector (FID) was set at 280°C.

### 3.3. GC-MS analysis

GC-MS analysis was carried on an Agilent-HP6890 gas chromatography system (same specifications and conditions as above), coupled with a high resolution Waters Micromass Autospec Ultima mass detector operating in the EI mode (70eV). Components identification was done by GC (according to RI) and GC-MS (according to fragmentation patterns) and by comparing results with literature [28,29,30]. Retention indices (RI) were calculated by comparing the retention times of the eluting peaks with those of standard hydrocarbon mixture using a Van Den Dool and Kratz formula [31]. The components concentration was calculated from GC peak areas without the use of correction factors.

## 4. Conclusions

This study revealed a high level of chemical polymorphism of the essential oils of *A. herba-alba* originated from different localities in southern Tunisia. Ten samples had a chemical composition similar to that published for *A. herba-alba* oils from different countries but eight samples had an original chemical composition. This remarkable polymorphism could be due to differences in the ecological and climatic characteristics of the localities from where samples were taken.
